# Supplementary material for: A brief questionnaire measure of multidimensional schizotypy predicts interview-rated symptoms and impairment
Source: PLoS One. 2020 Aug 10;15(8):e0237614. doi: 10.1371/journal.pone.0237614 (PMC7416934; doi:10.1371/journal.pone.0237614)
Supplement: S1 Table — (DOCX) [file pone.0237614.s001.docx]

**Supplementary Table 1. Bivariate Correlations of Cannabis Use and Interview Measures of Symptoms and Impairment.**

Correlation with

Outcome Measure Cannabis Use

Global Functioning -.13

SIPS-P total .05

SIPS-D total .16*

Negative Symptoms .00

NSM Attention  **.31*****

Schizotypal Symptoms -.05

Schizoid Symptoms -.06

Paranoid Symptoms .00

Any Cluster A Personality Disorder -.11

Never Dated **-.31*****

<2 Close Friends -.07

Mental Health Treatment .10

Major Depressive Episode -.01

Manic/Hypomanic Episode .01

Suicidal Ideation .04

**p* < .05 ***p* < .01 ****p* < .001

Medium effect sizes in bold
