# Supplementary material for: A brief questionnaire measure of multidimensional schizotypy predicts interview-rated symptoms and impairment
Source: PLoS One. 2020 Aug 10;15(8):e0237614. doi: 10.1371/journal.pone.0237614 (PMC7416934; doi:10.1371/journal.pone.0237614)
Supplement: S2 Table — (DOCX) [file pone.0237614.s002.docx]

**Supplementary Table 2. Linear Regressions Examining Prediction by the Multidimensional Schizotypy Scale-Brief Subscales and Cannabis Use (n = 177)**

|  | Step 1 | | | Step 2 | Step 3 | | |
| --- | --- | --- | --- | --- | --- | --- | --- |
|  | MSS-B Positive Schizotypy | MSS-B Negative Schizotypy | MSS-B Disorganized Schizotypy | Cannabis Use | Positive Schizotypy x Cannabis Use | Negative Schizotypy x Cannabis Use | Disorganized Schizotypy x Cannabis Use |
| Criteria: | β | β | β | β | β | β | β |
|  |  |  |  |  |  |  |  |
| Global Functioning | -.131** | -.455*** | -.375*** | -.124* | -.062 | .057 | .037 |
| SIPS-P total | .517*** | .254*** | .082 | .028 | -.030 | .028 | -.115 |
| SIPS-D total | .167** | .173*** | .571*** | .080 | -.075 | -.008 | -.101 |
| Negative Symptoms | -.117* | .617*** | .260*** | .038 | .067 | -.021 | .042 |
| NSM Attention | .124 | .056 | .514*** | .232*** | .015 | -.169* | .021 |
| Schizotypal Symptoms | .375*** | .437*** | .117 | -.049 | -.019 | -.018 | -.096 |
| Schizoid Symptoms | -.164** | ***.614****** | .015 | .017 | .049 | -.032 | .073 |
| Paranoid Symptoms | .188** | .289*** | .055 | .014 | -.058 | -.033 | -.059 |
|  |  |  |  |  |  |  |  |

**p* < .05 ***p* < .01 ****p* < .001

Each row represents a separate linear regression analysis in which the three MSS-B subscales were entered simultaneously at step 1, cannabis use rating was entered at step 2, and the schizotypy x cannabis interactions were entered simultaneously at step 3 as predictors of the quantitative interview measures
