# Supplementary material for: A brief questionnaire measure of multidimensional schizotypy predicts interview-rated symptoms and impairment
Source: PLoS One. 2020 Aug 10;15(8):e0237614. doi: 10.1371/journal.pone.0237614 (PMC7416934; doi:10.1371/journal.pone.0237614)
Supplement: S3 Table — (DOCX) [file pone.0237614.s003.docx]

**Table 3. Binary Logistic Regressions Examining Prediction by the Multidimensional Schizotypy Scale-Brief Subscales and Cannabis Use (*n* = 177)**

|  | **Step 1** | | | | | | **Step 2** | | **Step 3** | | | | | |
| --- | --- | --- | --- | --- | --- | --- | --- | --- | --- | --- | --- | --- | --- | --- |
| **Criteria:** | **MSS-B Positive Schizotypy** | | **MSS-B Negative Schizotypy** | | **MSS-B Disorganized Schizotypy** | | **Cannabis Use** | | **Positive Schizotypy x Cannabis Use** | | **Negative Schizotypy x Cannabis Use** | | **Disorganized Schizotypy x Cannabis Use** | |
|  | **Odds**  **Ratio** | **95% CI** | **Odds**  **Ratio** | **95% CI** | **Odds**  **Ratio** | **95% CI** | **Odds**  **Ratio** | **95% CI** | **Odds**  **Ratio** | **95% CI** | **Odds**  **Ratio** | **95% CI** | **Odds**  **Ratio** | **95% CI** |
| Never Dated | .98 | .70 – 1.36 | 1.34* | 1.02 – 1.77 | .86 | .62 – 1.18 | .37*** | .21 – 0.65 | 1.08 | .62 – 1.86 | .69 | .32 – 1.50 | .51 | .21 – 1.27 |
| <2 Close Friends | .69 | .35 – 1.36 | 1.99*** | 1.33 – 2.97 | .97 | .57 – 1.67 | .83 | .39 – 1.76 | 1.79 | .79 – 4.07 | 1.64 | .72 – 3.74 | 1.09 | .52 – 2.30 |
| Mental Health Trt | .99 | .72 – 1.34 | .92 | .69 – 1.23 | 1.76*** | 1.31 – 2.34 | 1.11 | .80 – 1.53 | .90 | .66 – 1.23 | .91 | .63 – 1.32 | .94 | .67 – 1.30 |
| Any Cluster A PD | .54 | .16 – 1.78 | 4.17*** | 1.85 – 9.44 | .35 | .05 – 2.44 | .33 | .01 – 8.64 | @ | @ | @ | @ | @ | @ |
| Major Depressive Ep. | 1.16 | .85 – 1.58 | 1.02 | .77 – 1.36 | 1.68*** | 1.27 – 2.24 | .87 | .61 – 1.23 | .81 | .57 – 1.16 | .91 | .63– 1.33 | .93 | .67 – 1.29 |
| Manic/Hypomanic Ep. | .98 | .52 – 1.84 | .94 | .53 – 1.69 | .87 | .46 – 1.64 | 1.06 | .55 – 2.01 | .97 | .50 – 1.86 | 1.49 | .71 – 3.09 | 1.29 | .60 – 2.79 |
| Suicidal Ideation | .84 | .57 – 1.23 | 1.36* | 1.01 – 1.83 | 1.81*** | 1.33 – 2.46 | 1.04 | .69 – 1.55 | .89 | .59 – 1.33 | .88 | .60 – 1.29 | .75 | .52 – 1.06 |

**p* < .05 ***p* < .01 ****p* < .001

@ model failed to converge for this step

Each row represents a separate binary logistic regression analysis in which the three MSS-B subscales were entered simultaneously at step 1, cannabis use rating was entered at step 2, and the schizotypy x cannabis interactions were entered simultaneously at step 3 as predictors h of the categorical interview measures
