# Supplementary material for: A brief questionnaire measure of multidimensional schizotypy predicts interview-rated symptoms and impairment
Source: PLoS One. 2020 Aug 10;15(8):e0237614. doi: 10.1371/journal.pone.0237614 (PMC7416934; doi:10.1371/journal.pone.0237614)
Supplement: Attachment — Submitted filename: Kemp PLOS response to reviewers.docx [file pone.0237614.s004.docx]

Summary of Revisions for “A Brief Questionnaire Measure of Multidimensional Schizotypy Predicts Interview-rated Symptoms and Impairment”, by Kemp, Bathery, Barrantes-Vidal, and Kwapil for publication in PLOS ONE.

Please note that we now indicate on page 10, lines 223 to 224 that the de-identified data for the study is available on Open Science Framework at: <https://osf.io/rky2d/>

We updated the in-press references that are now published. We moved the tables into the text.

We updated the manuscript to include the full name of the ethics committee/institutional review board(s) that approved our study (page 7, lines 159 to 160).

**Responses to Reviewer #1:**

*The authors describe a study in which they evaluate the correspondence between a new, brief measure of multidimensional schizotypy (MSS-B), interview-based ratings of schizotypy, and other related outcomes. They find the expected associations between dimensions of schizotypy as assessed with the MSS-B and the interview-based measures.*

*This an extremely clear, well-written, and straightforward paper that lends further support to a new, brief tool for assessing dimensions of schizotypy. I think it will make a nice contribution to the schizotypy literature. I have minimal comments for the authors.*

We appreciate the Reviewer’s comments about the clarity and potential contribution of the manuscript.

** Since the aim, hypotheses, methods, and results are so straightforward, I thought parts of the paper (e.g. the discussion) could be shorter.*

We appreciate the Reviewer’s recommendation and have deleted 114 words from the discussion section (see track changes for specific deletions).

** I’m curious as to why the authors assessed negative symptoms with the NSM as opposed to the negative symptom scores from the SIPS.*

We appreciate the Reviewer raising this point. Self-report and interview-based measures of negative symptoms and negative schizotypy often suffer from measurement problems including limited content coverage and saturation by unrelated constructs such as depression and neuroticism. The issue with depression is especially vexing. Depression and negative symptoms/schizotypy share some phenotypic similarities such as anhedonia, amotivation, and social disinterest. However, conceptually they appear to be distinct. Specifically, they are differentiated in that negative symptoms/schizotypy tend to be trait-like and enduring, and not characterized by markedly elevated negative affect. On the other hand, depression is more often episodic and characterized by negative affect. Our interview, questionnaire, and daily life studies have typically found minimal or no associations of negative symptoms with depression and neuroticism (and strong associations of negative and schizoid symptoms), but the selection of the measure of negative symptoms is crucial. For example, Barrantes-Vidal et al. (2013) found that the CAARMS negative symptom interview ratings were more strongly associated with depression and positive schizotypy than with negative schizotypy. The present study employed the NSM negative symptom assessment because previous Interview studies (e.g., Kwapil et al., 2008; Racioppi et al., 2018) demonstrated that the NSM is strongly related to negative schizotypy and to schizoid symptoms, provides comprehensive coverage of negative symptoms, and provides rigorous screening of unrelated conditions (e.g., depression) that could erroneously be rated as negative symptoms. Please note that we clarified this in the manuscript on page 9, lines 187 to 189:

The NSM was used because it provides comprehensive coverage of negative symptoms and, unlike other interview measures of negative symptoms, it does not appear to be highly saturated with depressive and positive symptoms [24].

** Could the authors comment on how the associations in the current manuscript compare to those with the full MSS described in Kemp et al.?*

We appreciate the Reviewer raising this point and apologize for not clearly addressing this (as it is an important focus of the present study!). We now indicate in the discussion section on page 22, lines 424 to 425 that:

The pattern of findings and effect sizes was nearly identical with the findings from Kemp et al, [20] for the full-length MSS.

Please note that in adding this information to the discussion section, we were mindful of the Reviewer’s previous recommendation that we shorten the discussion.

In addition, we specified in the abstract (line 38) that the present results for the MSS-B subscales are closely comparable to the findings for the full-length MSS in Kemp et al.

** I’m not sure I understand the third column of table 4 - how is that value calculated?*

We apologize for not clearly presenting the results in Table 4. We contend that schizotypy is a multidimensional construct with positive, negative, and disorganized dimension that have unique patterns of symptoms and impairment. Therefore, we recommend that studies should examine the unique associations of these dimensions rather than treating schizotypy as a homogenous construct and relying on a total schizotypy score when using measures such as the MSS-B. In order to empirically demonstrate this, we compared the variance accounted for in our quantitative outcome measures by the MSS-B total score (first column) with the proportion of variance accounted for by the three MSS-B subscales (second column). This proportion (variance accounted for by the MSS-B total divided by variance accounted for by the three subscales) is presented in the third column. Consistent with findings from interview (e.g., Kemp et al., in press), questionnaire (Kemp et al., 2020) and cognitive (Sahakyan et al., 2019) studies, the total MSS-B score only accounts for approximately ½ of the variance accounted for by the subscales (even though they are based on the same items). In order to clarify the value of the third column, we added the following sentence to page 17, lines 318 to 320:

The final column in the table shows how much variance the MSS-B total score accounts for relative to the total variance accounted for by the three MSS-B subscales.

We also added this information to Table 4.
